# Bax and Bak jointly control survival and dampen the early unfolded protein response in pancreatic β-cells under glucolipotoxic stress

**DOI:** 10.1038/s41598-020-67755-3

**Published:** 2020-07-03

**Authors:** Sarah A. White, Lisa S. Zhang, Daniel J. Pasula, Yu Hsuan Carol Yang, Dan S. Luciani

**Affiliations:** 1 Diabetes Research Group, BC Children’s Hospital Research Institute, Vancouver, BC Canada; 2 University of British Columbia, Vancouver, BC Canada; 3 BC Children’s Hospital Research Institute, A4-183, 950 W. 28th Avenue, Vancouver, BC V5Z 4H4 Canada

**Keywords:** Stress signalling, Apoptosis

## Abstract

ER stress and apoptosis contribute to the loss of pancreatic β-cells under pro-diabetic conditions of glucolipotoxicity. Although activation of canonical intrinsic apoptosis is known to require pro-apoptotic Bcl-2 family proteins Bax and Bak, their individual and combined involvement in glucolipotoxic β-cell death are not known. It has also remained an open question if Bax and Bak in β-cells have non-apoptotic roles in mitochondrial function and ER stress signaling, as suggested in other cell types. Using mice with individual or combined β-cell deletion of Bax and Bak, we demonstrated that glucolipotoxic β-cell death in vitro occurs by both non-apoptotic and apoptotic mechanisms, and the apoptosis could be triggered by either Bax or Bak alone. In contrast, they had non-redundant roles in mediating staurosporine-induced apoptosis. We further established that Bax and Bak do not affect normal glucose-stimulated β-cell Ca^2+^ responses, insulin secretion, or in vivo glucose tolerance. Finally, our experiments revealed that combined deletion of Bax and Bak amplified the unfolded protein response in islets during the early stages of chemical- or glucolipotoxicity-induced ER stress. These findings shed new light on roles of the core apoptosis machinery in β-cell survival and stress signals of importance for the pathobiology of diabetes.

## Introduction

In the pathogenesis of obesity-associated type 2 diabetes, chronic exposure to elevated blood glucose concentrations (glucotoxicity) and excess levels of circulating lipids (lipotoxicity) promote progressive failure and death of the insulin-secreting pancreatic β-cells^1–3^. Moreover, the combined excess of glucose and lipids (glucolipotoxicity) may synergize to cause a faster and more severe progression of β-cell demise^4,5^. Endoplasmic reticulum (ER) stress contributes to the loss of functional β-cells^1,6–8^. ER stress activates the unfolded protein response (UPR), an adaptive measure initially aimed at restoring homeostasis through down-regulation of general protein translation and up-regulation of select genes encoding molecular chaperones and the machinery for ER associated degradation of proteins (ERAD). If the UPR fails to mitigate ER stress, a transition occurs whereby the UPR instead promotes apoptosis^9^. While the involvement of ER stress in glucose- and lipid-induced β-cell pathobiology is widely accepted, many details regarding the regulation of the β-cell UPR, and its mechanistic links to β-cell death, remain unclear.

Chronic cellular stress triggers cell death by intrinsic apoptosis, which is regulated by proteins in the Bcl-2 family^10^. Mitochondrial outer membrane permeabilization, a critical step in this process, is carried out by the pro-apoptotic family members Bax and Bak^11,12^. Combined deletion of Bax and Bak thus provides a powerful means of dissecting the involvement of the canonical intrinsic apoptosis pathway. Unrelieved ER stress has also been demonstrated to trigger Bax and Bak-dependent death of several cell types^11,13–15^. However, most studies of Bax and Bak in primary pancreatic β-cells under ER stress, including in human type 2 diabetes, have been correlative reports of increased expression or mitochondrial translocation of Bax^8,16,17^. The few loss-of-function studies that have specifically addressed their requirement for β-cell apoptosis suggest that loss of either protein alone provides partial protection from cytokine-induced death^18^, and that Bax^−/−^ β-cells show a modest protection from glucolipotoxicity^19^. This could reflect significant overlap in signaling β-cell apoptosis, not unlike their redundant roles in cell death during development^20^. However, apoptosis induced by chronic glucotoxicity or Pdx1 deficiency appears to preferentially engage Bax^21,22^. This indicates that the relative involvement of the two proteins may be context- and stress-specific, but to date no study has included a full comparison of the individual and combined contributions of both Bax and Bak to stress-induced death in primary β-cells.

A growing body of work is also uncovering physiological functions of various Bcl family apoptosis proteins^23–32^. We have reported that anti-apoptotic Bcl-2 and Bcl-x_L_ modulate β-cell mitochondrial metabolism, glucose responsiveness, and ROS signaling^30,31^. Others have shown that glucokinase activity is controlled by pro-apoptotic Bad^26,27^. Studies in non-β-cells have implicated both Bax and Bak in ER physiology and UPR signaling^13,28^, and Bax in the regulation of mitochondrial bioenergetics^29^. In addition to their respective functions in stress-induced death, it therefore also remains an important question if Bax and Bak have non-apoptotic functions in pancreatic β-cells.

In this study we established lines of inducible knockout mice to determine the effects of single and combined Bax-Bak deficiency on β-cell function, ER stress signaling, and survival. We provide the first direct molecular assessment of the requirement for the intrinsic, mitochondrial, apoptosis pathway in glucolipotoxic β-cell death. Moreover, our data reveal that Bax and Bak do not play significant roles in β-cell function, but dampen the UPR in pancreatic islets during the early stages of ER stress.

## Results

### Loss of Bax and/or Bak does not affect normal islet function and glucose homeostasis

**Figure 1:**
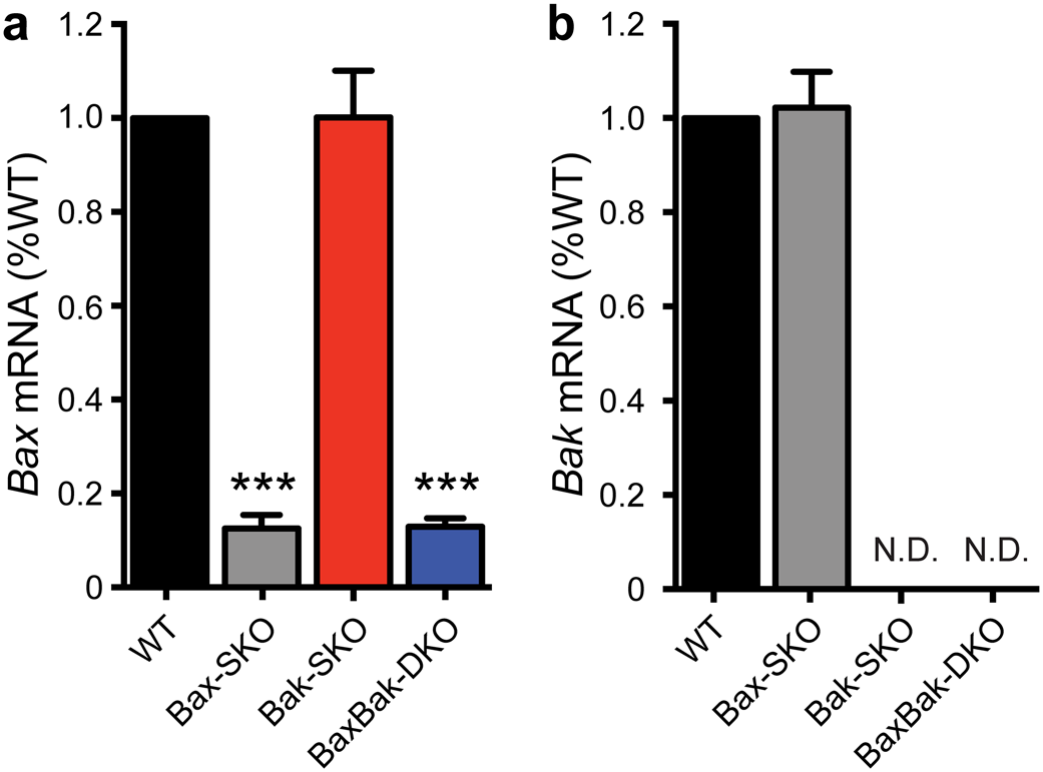
Combined and individual knockout of Bax and Bak in pancreatic islets. (**a**) Relative *Bax* and (**b**) relative *Bak* mRNA levels after tamoxifen administration and Cre recombination in islets from Bax SKO (Bax^flox/flox^:Bak^+/+^:Cre^+^), Bak SKO (Bax^flox/flox^:Bak^−/−^), and Bax-Bak DKO (Bax^flox/flox^:Bak^−/−^:Cre^+^) mice compared to WT (Bax^flox/flox^:Bak^+/+^) islets. (n = 4–6 animals of each genotype; islets isolated from 12 to 16 week old mice 1-week post-tamoxifen administration). Data are normalized to WT and represent mean ± SEM. Statistical comparisons were done using 1-way ANOVA with Bonferroni post-hoc tests. ***p < 0.001 compared to WT; N.D. not detected.

In mice, combined global knockout of Bax and Bak results in severe developmental abnormalities and perinatal death of most double knockout animals^20^. Tissue-specific gene deletion is therefore required to study the effect of combined Bax and Bak loss. We previously established a line of mice that are null for Bak in all tissues and have additional tamoxifen (TM)-inducible deletion of Bax in Pdx1-expressing islet-cells, which includes all β-cells^30^. To allow a comprehensive study of the individual and combined roles of Bax and Bak, we have now expanded this mouse colony to include the four genotypes: Wild-type (WT), Bax single-knockout (Bax SKO), Bak single knockout (Bak SKO), and Bax-Bak double knockout (Bax-Bak DKO) (cf. Methods for details). Using real-time PCR we confirmed the complete absence of Bak mRNA, as well as > 80% TM-induced knockdown of Bax mRNA in islets from mice carrying the floxed Bax alleles (Fig. 1). The latter agrees with Cre-induced gene deletion in β-cells and possibly a small number of islet δ-cells that express Pdx1. We previously demonstrated by western blot that the decreases in Bax and Bak mRNA are associated with similarly reduced islet protein^30^.

**Figure 2:**
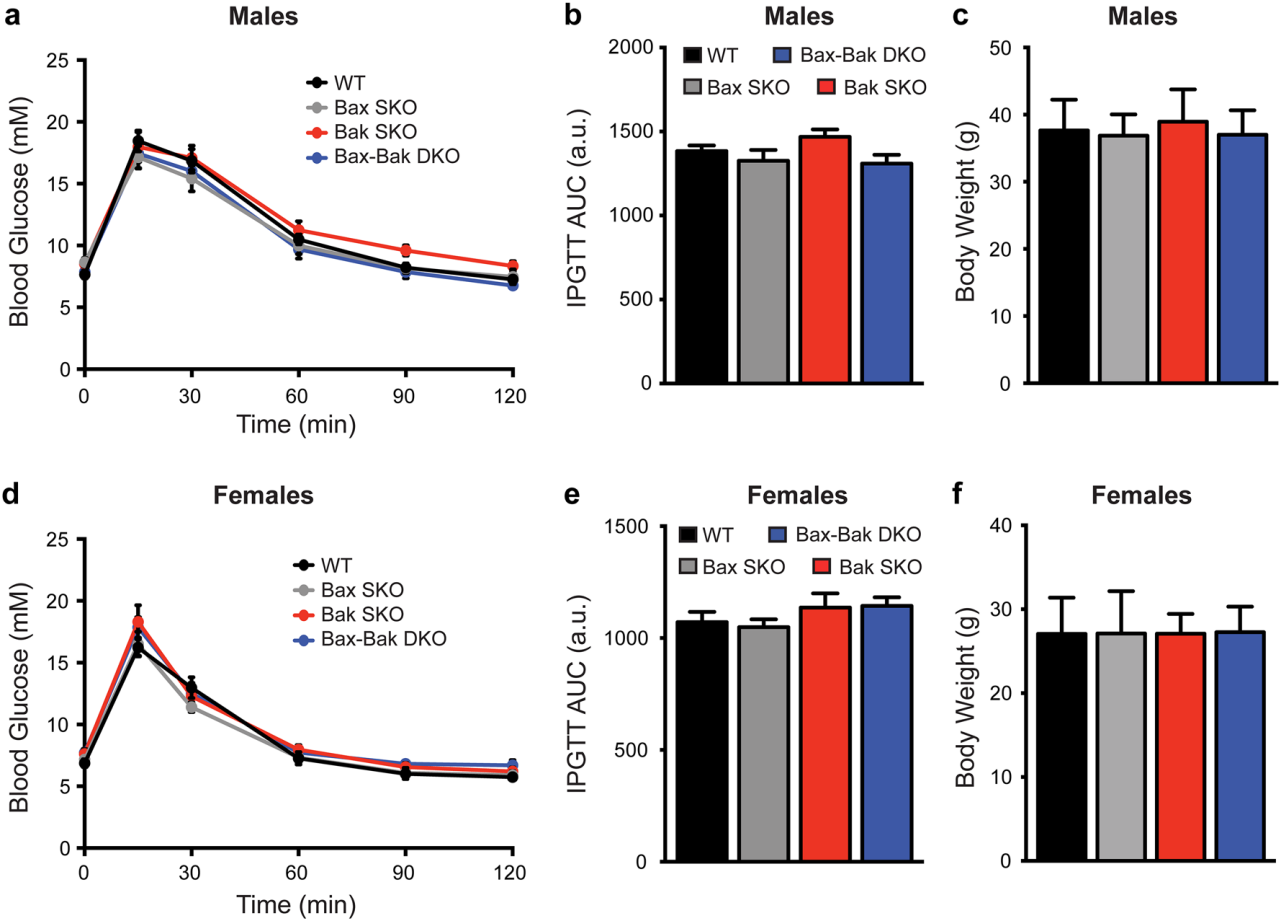
Loss of islet Bax and/or global Bak deletion does not affect body weight or in vivo glucose tolerance. Intraperitoneal glucose tolerance tests (IPGTT; 2 g/kg) and body weight analyses were performed in WT, Bax SKO, Bak SKO, and Bax-Bak DKO mice at 12–16 weeks of age. IPGTT blood glucose kinetics, area under the curve (AUC) of IPGTT profiles, and body weights, were not different between genotypes in male (**a**–**c**) and female (**d**–**f**) animals (n = 15–30 animals of each genotype in each sex for body weight data; n = 7–12 animals of each genotype in each sex for IPGTT data). Data represent mean ± SEM. a.u. arbitrary units. Statistical comparisons were done using 1-way ANOVA with Bonferroni post-hoc tests.

**Figure 3:**
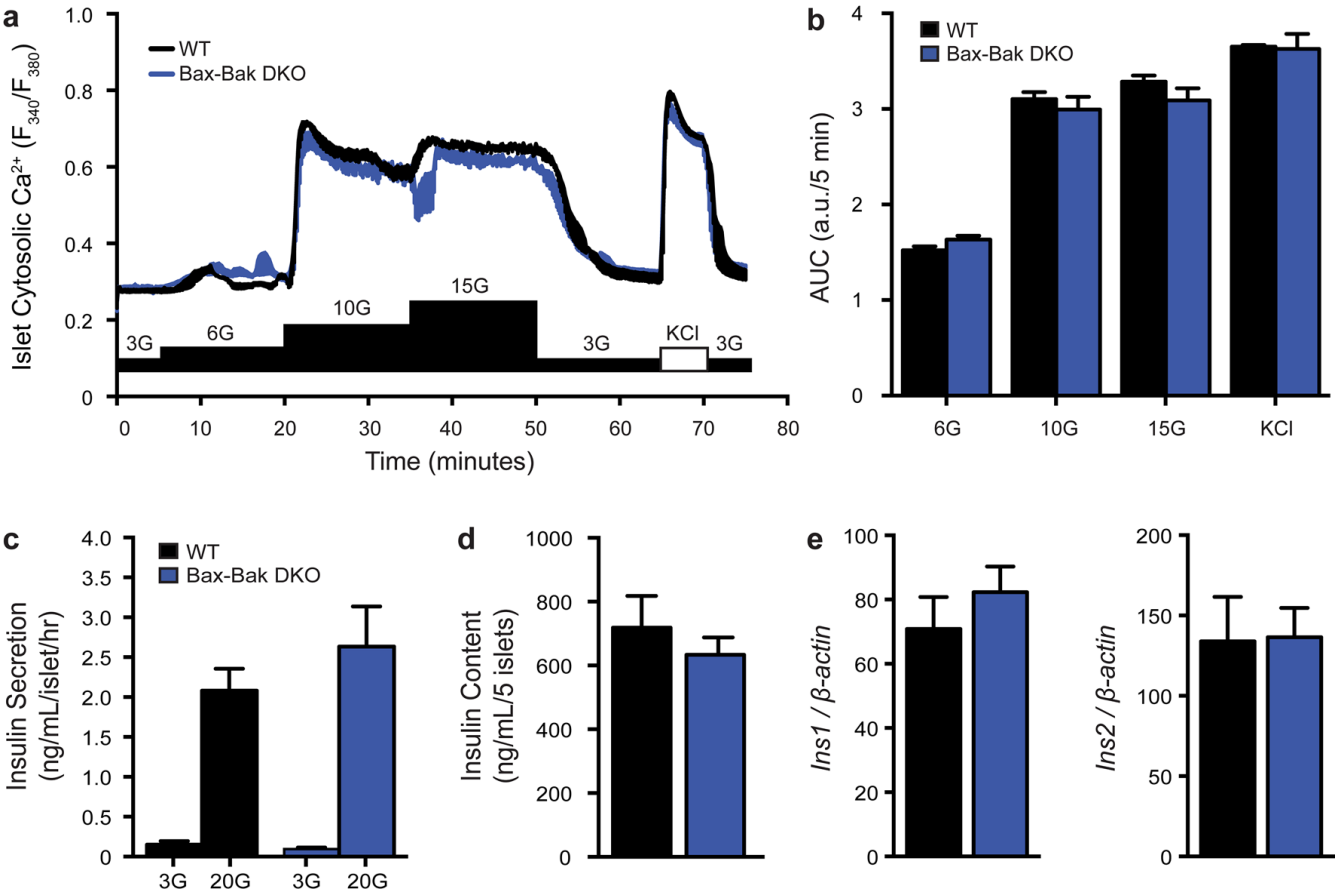
Loss of islet Bax and/or global Bak deletion does not affect islet physiology in vitro. (**a**) Average cytosolic calcium (Ca^2+^) responses of whole islets from WT and Bax-Bak DKO mice stimulated sequentially with increasing glucose concentrations (G = mM glucose) and 3 mM glucose + 30 mM KCl (KCl). (**b**) Area under the curve (AUC) analysis of the response to each stimulus in panel a. The AUC is expressed per 5 min intervals for comparison (n = 4 independent islet preparations). (**c**) Glucose stimulated insulin secretion from static incubations of isolated WT and Bax-Bak DKO islets subjected to sequential stimulation for 1 h each with 3 mM glucose (3G) and 20 mM glucose (20G) (n = 7 independent experiments). (**d**) Insulin content of size-matched islets from WT and Bax-Bak DKO mice (n = 5 independent experiments). (**e**) *Ins1* and *Ins2* mRNA expression in WT and Bax-Bak DKO islets normalized to *β-Actin* housekeeping gene (n = 4). Data represent mean ± SEM, a.u. arbitrary units. (**b**,**c**) were analyzed by 2-way ANOVA with Bonferroni post-hoc comparisons. (**d**,**e**) were analyzed by unpaired student’s t-tests.

Comparison of male and female mice of all four genotypes showed that single and double knockout mice all had normal body weights. Intraperitoneal glucose tolerance tests further demonstrated that there were no differences in the glucose tolerance of mice of all genotypes (Fig. 2). To more directly examine the impact of Bax-Bak deletion on β-cell function, we measured cytosolic Ca^2+^ responses and insulin secretion in isolated islets. No differences were observed in the cytosolic Ca^2+^ levels of WT and Bax-Bak DKO islets under basal conditions, or in the response to a stepwise glucose increase or direct depolarization with KCl (Fig. 3a,b). Double knockout of Bax and Bak did not affect islet insulin secretion under basal or acute glucose-stimulated conditions (Fig. 3c), and both islet insulin mRNA levels and insulin content did not differ between WT and Bax-Bak DKO islets in normal culture (Fig. 3d,e). Single deletion of Bax or Bak also did not affect islet function (data not shown). These results demonstrate that individual or combined loss of Bax and/or Bak in the adult β-cell has no detectable effects on the function of pancreatic islets under normal, non-stressed, conditions. Further, our comparison of the four different genotypes allows us to conclude that the life-long loss of Bak in all tissues does not affect in vivo glucose homeostasis.

### Single and double Bax/Bak deletion reveals non-redundant roles in STS-induced β-cell apoptosis

**Figure 4:**
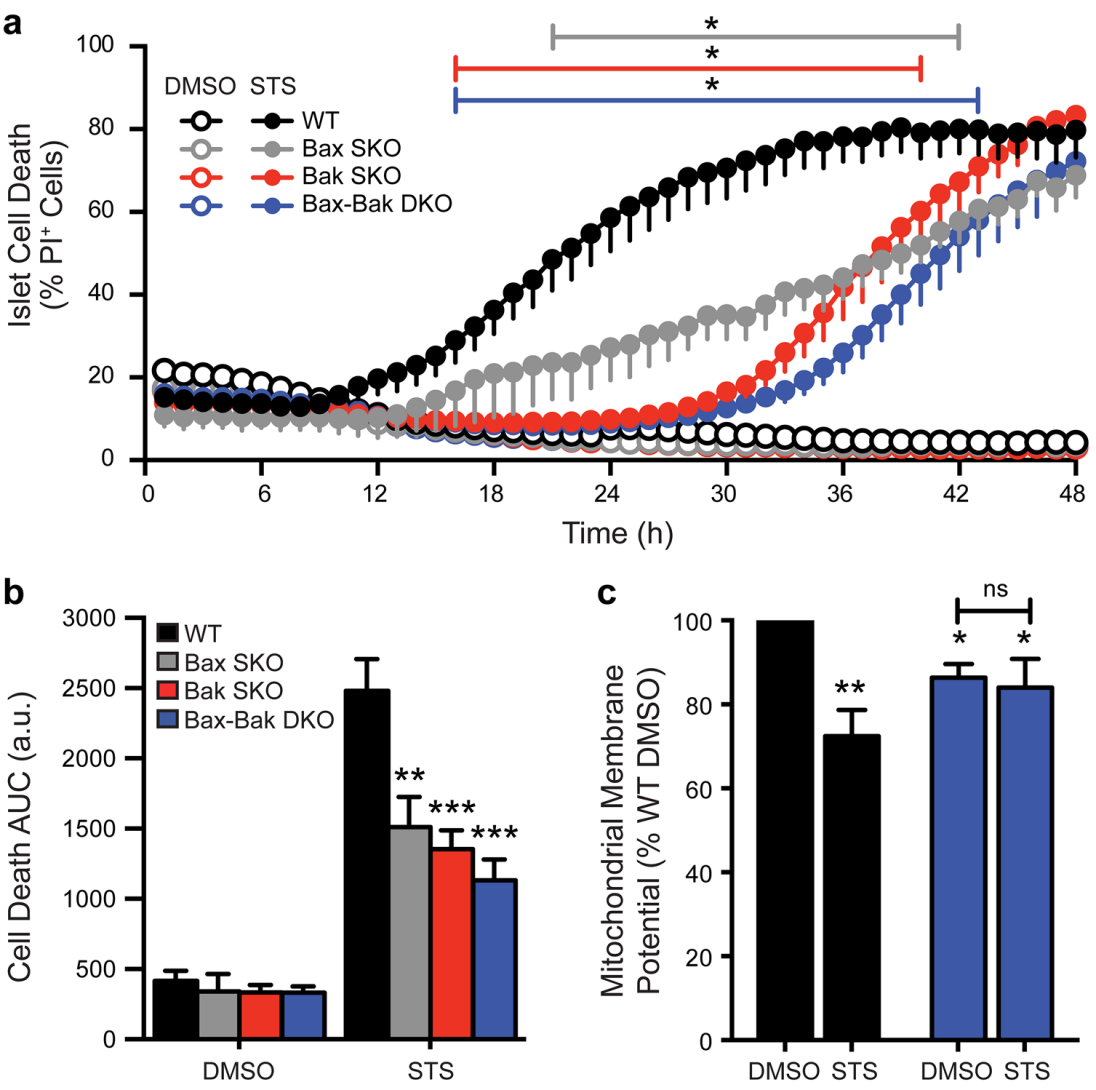
Relative contribution of Bax and Bak to staurosporine-induced mitochondrial apoptosis. (**a**) Time-course cell death analysis of dispersed islet cells from WT (black), Bax SKO (gray), Bak SKO (red), and Bax-Bak DKO (blue) mice. Cells were treated with 1 μM staurosporine (STS; filled circles) or DMSO control (open circles) for 48 h. The percentage of dead cells was measured as the number of propidium iodide positive (PI^+^) cells relative to total number of cells in each experiment. Coloured lines above the graphs indicate the time ranges where *p < 0.05 for the three knockout genotypes compared to WT. (**b**) Area under the curve (AUC) analysis of the cell death profiles in panel a, **p < 0.01 and ***p < 0.001 compared to WT (n = 4–11 independent islet cell preparations for the various genotypes). (**c**) Mitochondrial membrane potential (Mean TMRE fluorescence intensity) of WT and Bax-Bak DKO islet cells treated with DMSO control or 1 μM STS for 24 h. Data are normalized to WT DMSO control. *p < 0.05 and **p < 0.01 compared to WT DMSO (n = 4 independent islet cell preparations). Data represent mean ± SEM, a.u. arbitrary units*.* Statistical comparisons were done using 2-way ANOVA with Bonferroni post-hoc tests.

To investigate the relative importance of Bax and Bak in executing β-cell apoptosis, we first performed a detailed kinetic analysis of staurosporine (STS)-induced death. STS is a pan-kinase inhibitor that has been demonstrated to activate Bcl-2-sensitive, Bax-Bak-dependent, apoptosis^11,33^. Analysis of STS-induced death in islet cells of all four genotypes showed that WT cell death was initiated after roughly 12 h and reached a maximal plateau by 36 h (Fig. 4a). Absence of Bax and/or Bak markedly prevented cell death, with their combined deletion providing the highest degree of protection (Fig. 4a,b). Overall, the onset of STS-induced death of Bax SKO islet cells was delayed by several hours and then progressed at a significantly reduced rate. No death of Bak SKO and Bax-Bak DKO cells was detected until after more than 24 h of STS-induced stress. That Bax SKO islet cells were less protected than those from Bak null mice likely reflects death of the islet cells that do not express Cre and therefore still have Bax. Consistent with loss of mitochondrial integrity in STS-induced apoptosis, WT islet cells showed a significant reduction in mitochondrial membrane potential after 24 h. In contrast, the mitochondrial polarization of Bax-Bak DKO β-cells did not change, further supporting that mitochondrial outer membrane permeabilization was blocked at this time-point (Fig. 4c). Notably, islet cell death reached similar levels in all genotypes after approximately 48 h of exposure to STS (Fig. 4a), indicating the activation of late-stage death that proceeds independently of the canonical machinery for intrinsic apoptosis.

Together, these findings demonstrate that the combined functions of Bax and Bak are required for STS-induced apoptosis in pancreatic β-cells, revealing that they cannot compensate for each other in this context. Further, our results show that chronic in vitro β-cell stress can eventually activate alternate, non-apoptotic, death mechanisms.

### Combined deletion of Bax and Bak is required for protection, and reveals multiple β-cell death modes, under glucolipotoxic stress

**Figure 5:**
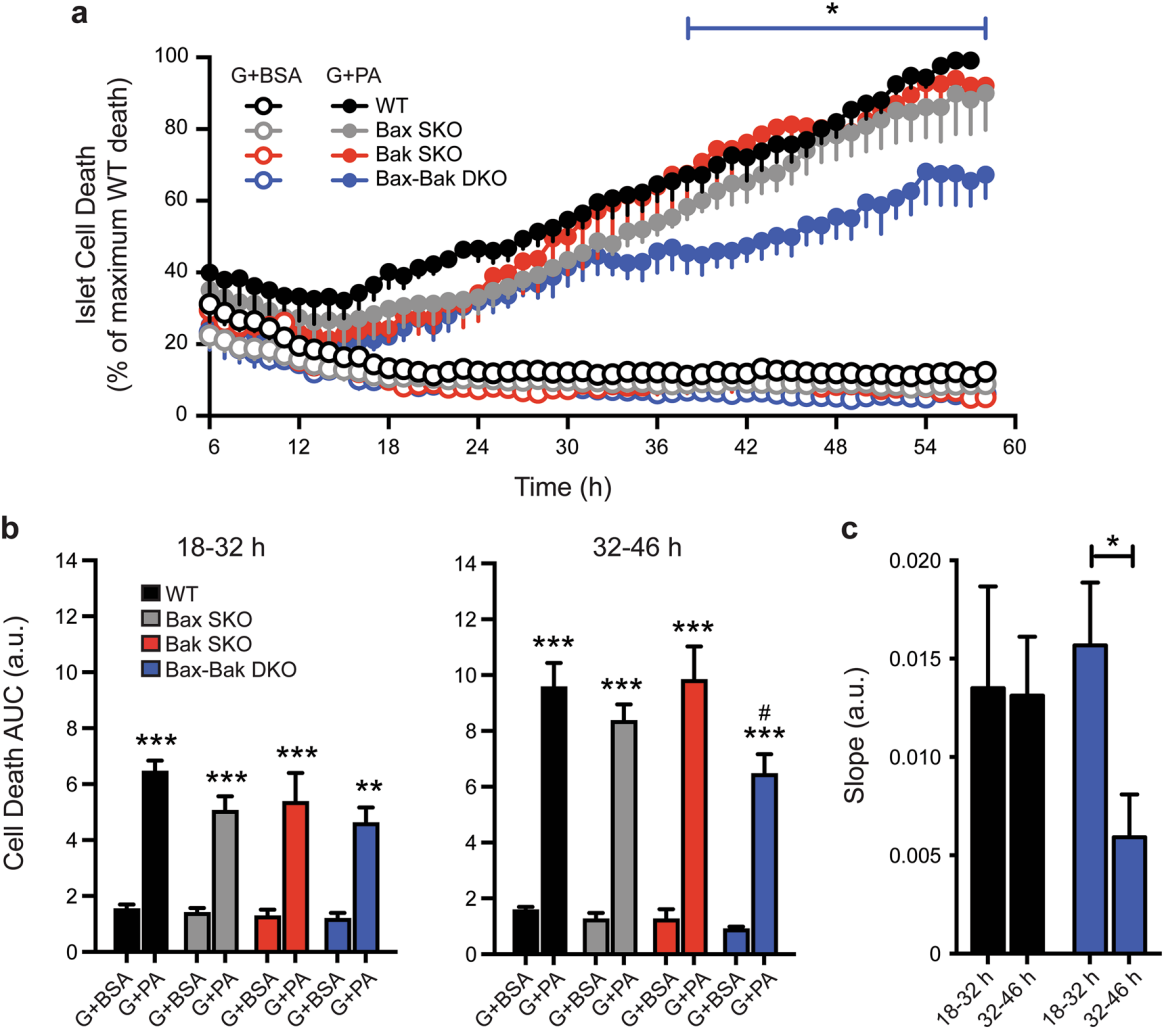
Combined knockout of Bax and Bak is required for significant protection against glucolipotoxicity. (**a**) Time-course analysis of cell death (PI incorporation) in cultures of dispersed WT, Bax SKO, Bak SKO, and Bax-Bak DKO islet cells treated with 25 mM glucose + BSA (G + BSA; open circles) or 25 mM glucose + 1.5 mM palmitate (G + PA; closed circles) for 58 h. Between experiments the degree of glucolipotoxic death after 58 h showed some variability (15–25% between islet cell preparations), but was always lowest in the Bax-Bak DKO cells. To facilitate analysis, the islet cell death was expressed relative to that in the WT cultures at the end of each individual experiment (100%). The blue line above the graph indicates the time range where *p < 0.05 for Bax-Bak DKO cell death compared to WT, Bax SKO, and Bak SKO cell death. (**b**) Area under the curve (AUC) analysis of islet cell death profiles in panel a comparing all genotypes and treatment conditions during two 14 h intervals representing early (18–32 h) and late (32–46 h) stages of cell death. **p < 0.01 and ***p < 0.001 comparing G + PA treatment to their respective G + BSA controls. ^#^*p* < 0.05 for Bax-Bak DKO G + PA treatment compared to WT G + PA treatment. (**c**) Rates of WT and Bax-Bak DKO islet cell death were compared by calculating the slope of cell death profiles in panel a for the early (18–32 h) and late (32–46 h) stages. *p < 0.05 (n = 4 independent islet cell preparations). Data represent mean ± SEM, a.u. arbitrary units*.* Statistical comparisons were done using 2-way ANOVA with Bonferroni post-hoc tests.

We next examined the requirement for Bax and Bak in mediating β-cell death during the diabetes-relevant conditions of glucose- and lipid-induced stress. Dispersed islet cells were cultured for up to 58 h in the presence of varying levels of glucose with or without addition of the free fatty acid palmitate. Culture in low glucose (5 mM glucose media with BSA), high glucose (25 mM glucose media with BSA), or under strong lipotoxic conditions alone (5 mM glucose with 1.5 mM palmitate in 6:1 ratio with BSA), did not result in detectable death of islet cells from WT, Bak SKO, Bax SKO, or Bax-Bak DKO mice (Fig. 5 and data not shown). In contrast, glucolipotoxic stress caused progressive death of islet cells of all four genotypes (Fig. 5). Only the combined knockout of Bax and Bak provided significant protection against the glucolipotoxic insult (Fig. 5a,b). Although this cell death assay does not distinguish between islet cell types, the results likely reflect changes primarily in the β-cell population, as pancreatic α-cells are highly resistant to (gluco)lipotoxicity-induced death^34^.

Inspection of the kinetic cell death profiles suggested an inflection point specifically in the curve of Bax-Bak DKO cells at approximately 32 h. Quantitation of the cell death during 14 h intervals before and after this time-point (Fig. 5b) showed a clear protection of Bax-Bak DKO cells during the later time-period (32–46 h). Slope analysis of the time-series further showed that the rate of death of WT and Bax-Bak DKO islet cells was similar at first and then decreased significantly in Bax-Bak DKO islet cells only (Fig. 5c). Intriguingly, the comparison of all four genotypes further indicates that either Bax or Bak alone is sufficient to signal this late-stage islet cell apoptosis (i.e. they are functionally redundant), which is in contrast to their combined requirement for STS-induced apoptosis.

### Bax and Bak dampen the early unfolded protein response in islets under ER stress

**Figure 6:**
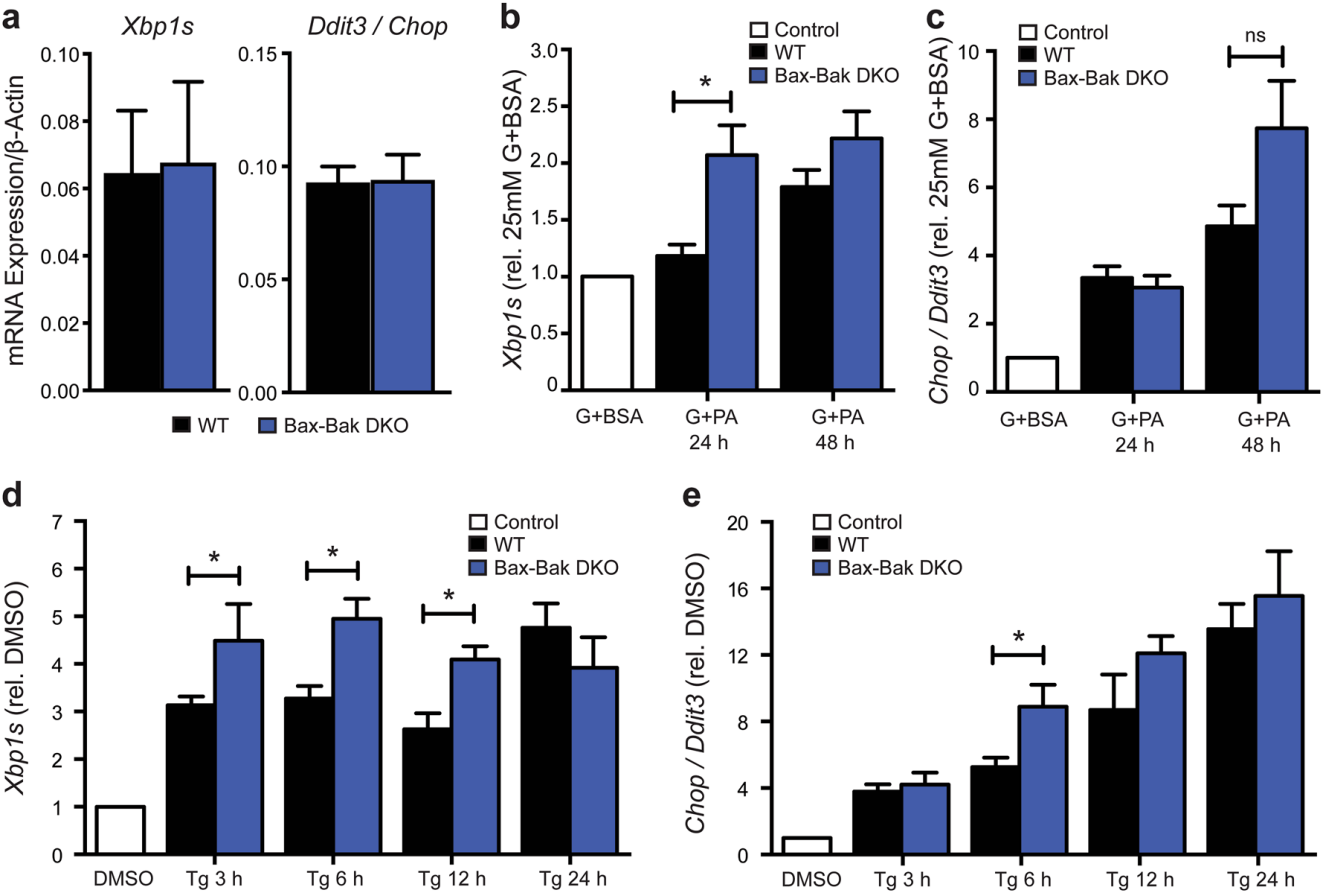
Double knockout of Bax and Bak augments early UPR signaling in islets under ER-stress. (**a**) Quantification of basal *Xbp1s* and *Chop* expression in untreated WT and Bax-Bak DKO islets. Quantification of the time-dependent expression of *Xbp1s* (**b**) and *Chop* (**c**) mRNA in WT and Bax-Bak DKO islets treated with 25 mM glucose + 1.5 mM palmitate (G + PA) or 25 mM glucose + BSA control (G + BSA) for 24 and 48 h. G + PA results are normalized against their respective control conditions for each time-point (represented by the white bar; n = 3–7 for each time-point). Quantification of the time-dependent expression of X*bp1s* (**d**) and *Chop* (**e**) in WT and Bax-Bak DKO islets treated with 100 nM Thapsigargin (Tg) compared to DMSO vehicle control (white control bar; n = 4–16 for each time-point). Data represent mean ± SEM. *p < 0.05; ns = not significant. Statistical comparisons were done using 1-way ANOVA with Bonferroni post-hoc tests.

Chronic hyperglycemia and elevated free fatty acids disrupt ER homeostasis and trigger ER stress, which contributes to β-cell dysfunction and death in diabetes pathogenesis. Motivated by evidence for involvement of Bcl-2 family proteins in the control of ER physiology^13,28^, we next investigated whether Bax and Bak have roles in regulating β-cell UPR signaling under glucolipotoxic conditions. During ER stress, expression of the transcription factor *Ddit3* (*Chop*) and a transcriptionally active form of *Xbp1* (*Xbp1s*) increase. *Chop* is up-regulated as a result of Perk activation and *Xbp1s* is up-regulated by increases in the transcription of total *Xbp1* and/or the splicing of *Xbp1* by Ire1α^35^. Under normal culture conditions WT and Bax-Bak DKO islets had similar expression levels of both *Chop* and *Xbp1s* (Fig. 6a), indicating that Bax and Bak are not required for maintenance of basal islet ER homeostasis. Relative to culture in high glucose alone, the combination of high glucose and palmitate time-dependently increased *Chop* and *Xbp1s* mRNA in islets of all 4 genotypes (Fig. 6b,c and data not shown). Compared to WT, *Xbp1s* transcripts were induced at significantly higher levels in Bax-Bak DKO islets after 24 h, but this difference evened out by 48 h (Fig. 6b). A trend toward higher *Chop* mRNA was also seen in DKO islets after 48 h, but this did not reach statistical significance (Fig. 6c). Single deletion of Bax or Bak did not result in any detectable differences compared to WT (data not shown). This suggests that Bax and Bak individually are sufficient to dampen the UPR in pancreatic islets under glucolipotoxic stress.

A glucolipotoxic insult activates a multifaceted β-cell response characterized not only by ER stress, but also by oxidative stress and general perturbations in other organelles^5^. We therefore also examined the UPR following more specific ER stress, induced by thapsigargin, a chemical inhibitor of SERCA-mediated ER Ca^2+^ uptake^36^. As expected, thapsigargin induced time-dependent expression of *Xbp1s* and *Chop* mRNA in the islets, with significant increases after just 3 h (Fig. 6d,e). Similar to what was seen during glucolipotoxic stress, thapsigargin-mediated induction of *Xbp1s* and *Chop* mRNA levels were accelerated as a result of Bax-Bak double deletion, and the difference evened out following more chronic stress. Together, these data suggest that Bax and Bak dampen earlier stages of β-cell ER stress signaling and the UPR.

**Figure 7:**
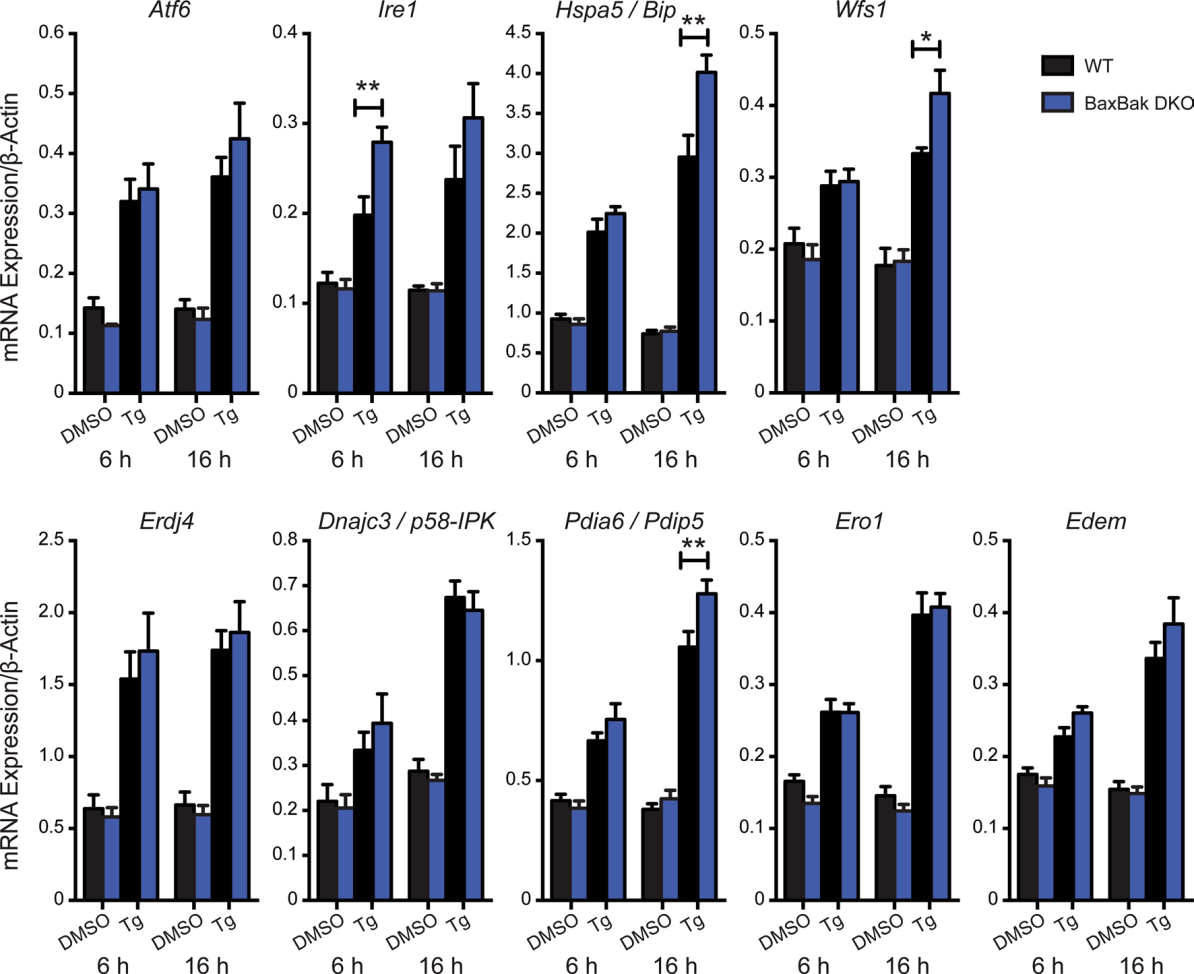
Downstream UPR gene expression in WT and Bax-Bak DKO islets under ER-stress. Gene expression analysis of UPR-inducers and downstream UPR target genes in WT and Bax-Bak DKO islets treated 6 or 16 h with 100 nM Thapsigargin (Tg) or DMSO vehicle control. Gene expression was normalized to the housekeeping gene *β-Actin*. Data represent mean ± SEM. *p < 0.05 and **p < 0.01; each transcript was analyzed by 2-way ANOVA with Bonferroni post-hoc tests (n = 6 independent islet preparations).

To further assess the impact of combined Bax-Bak deletion on β-cell UPR signaling we examined the time-dependent expression of other UPR genes, including molecular chaperones and downstream target genes involved in the regulation of ER redox status and protein degradation. Thapsigargin-induced ER stress increased the expression of all genes examined (Fig. 7). In no instances did the basal expression of the UPR-related genes differ between islets from WT and Bax-Bak DKO mice, and under none of the tested conditions were UPR-related transcripts higher in the WT islets. However, in Bax-Bak DKO islets the mRNA levels of *Ire1* itself were significantly increased after 6 h of treatment, and at 16 h the mRNA levels of the chaperone *Hspa5/Bip*, the ER-associated glycoprotein *Wfs1* (Wolframin)^37^, and the protein disulfide isomerase *Pdia6/Pdip5*, were significantly higher compared to WT islets (Fig. 7).

These findings demonstrate that Bax and Bak together attenuate the UPR in pancreatic islets, revealing modulatory functions in β-cell stress signaling prior to activation of mitochondrial outer membrane permeabilization.

## Discussion

In this study we used conditional and inducible gene deletion to dissect the roles of the apoptotic executioner proteins Bax and Bak in pancreatic β-cell function, as well as β-cell ER stress and death following combined exposure to high levels of glucose and fatty acids.

There is compelling evidence that apoptosis contributes to the loss of β-cell mass in humans with type 2 diabetes^3^. In line with this, Bax mRNA and protein levels are significantly increased in islets from patients with type 2 diabetes compared to non-diabetic controls^8,17^. Islet levels of both Bax and Bak also increase during the progression of type 2 diabetes in the db/db mouse, but not in islets from obese ob/ob mice that remain normoglycemic^38^. This suggests that Bax and Bak actively contribute to the diabetic islet phenotype. Accordingly, in vitro work has demonstrated roles for Bax, Bak, and other Bcl family members in β-cell death during lipotoxic and glucotoxic stress^16,17,21,39,40^.

We did not detect islet cell death under culture with high glucose alone, or in response to palmitate in the presence of low glucose. This supports the idea that elevated glucose and lipids together induce a more detrimental ‘glucolipotoxic’ state^5,41^. Others have demonstrated that glucotoxic β-cell death is Bak-independent, but involves Bax^21^, likely via ‘upstream’ pro-apoptotic BH3-only family members Puma and Bim^12,17^. This was observed using longer and more severe glucotoxic conditions than those we used here, and therefore does not disagree with our data. Lipotoxicity is known to activate Bax and β-cell apoptosis^1,16^. The resilience of our primary mouse cells to pure lipotoxicity prevented us from clarifying the role of Bak in lipotoxic β-cell death.

Bax-Bak redundancy characterizes apoptosis during development^20^ and instances of pathogenic cell death in vivo^28,42^. In this study, we found that primary islet cell death during the compound stress of glucolipotoxicity progressed normally with either Bax or Bak alone, suggesting a similar redundancy. In contrast, STS-induced β-cell apoptosis required the presence of both proteins. In conjunction with studies that report preferential involvement of Bax in β-cell apoptosis under glucotoxicity^21^ and Pdx1 deficiency^22^, this illustrates that the relative requirement for Bax and Bak in β-cell death can depend on the specific form of stress.

Our Bax-Bak double knockout model allowed us to identify both apoptotic and non-apoptotic cell death during STS treatment and glucolipotoxicity. This agrees with the finding that stressed primary β-cells often die without all the morphological features of apoptosis^43^. Moreover, death of Pdx-1-deficient β-cells happens by apoptosis, as well as necrosis involving the mitochondrial permeability transition pore^44–46^. We observed late-stage death of STS-treated Bax-Bak DKO β-cells that reflect an apoptosis-to-necrosis switch. That a combination of high glucose and palmitate could kill β-cells of all Bax-Bak genotypes shows that glucolipotoxicity also triggers both apoptotic and non-apoptotic pathways. Under these conditions, our analyses provide some indications that Bax- and Bak-independent, i.e. non-apoptotic, mechanisms may dominate in the earlier stages of glucolipotoxic stress and then shift toward more Bax- and Bak-mediated apoptosis. A transition from necrotic to apoptotic cell death has previously been suggested by fluorescence cytotoxicity assays in lipotoxic cultures of rat islet cells^47^ and our results provide genetic evidence to support such a transition. Further studies are warranted to clarify the molecular nature, and putative crosstalk, of apoptotic and non-apoptotic β-cell loss under various stresses. It will also be important to determine the extent to which our single-cell results are recapitulated in intact islets and in vivo*,* where important signals from cell–cell contacts, as well as autocrine and paracrine factors are not disrupted. Insights into the mechanisms of β-cell death may help identify therapies to prevent their loss in diabetes. However, it should be considered if preventing the execution of dying β-cells in vivo may instead cause build-up of cells that are stressed and dysfunctional. This could possibly even exacerbate the disease, as exemplified by stress-induced accumulation of senescent β-cells in models of both type 1 and type 2 diabetes^48,49^.

Glucose-stimulated insulin release from β-cells depends on mitochondrial oxidative metabolism. A significant pool of cellular Bak is anchored in the outer mitochondrial membrane. In non-apoptotic cells this Bak binds to voltage-dependent anion channel 2, a protein involved in the mitochondrial transport of ions and metabolites^50^. Furthermore, Bax and Bak have both been implicated in the control of normal mitochondrial fusion processes^51,52^. This all hints that Bax and Bak might affect mitochondrial physiology. Indeed, Bax has been shown to promote mitochondrial bioenergetics in healthy HCT-116 cells and hepatocytes^29^. Under normal culture conditions we observed a modest reduction in the mitochondrial membrane potential of Bax-Bak DKO islet cells, relative to WT. However, we established that this difference did not significantly alter glucose-stimulated Ca^2+^ signals and insulin secretion, which is further backed by normal glucose homeostasis in our knockout mice. In contrast, we and others have demonstrated that anti-apoptotic Bcl-2 and Bcl-x_L_ dampen β-cell glucose responses, and that this is independently of Bax or Bak^30,53^. Also, pro-apoptotic Bad modulates insulin secretion via interactions with glucokinase^27^. It thus appears that Bcl-2 family proteins show different degrees of involvement in β-cell mitochondrial physiology.

Pools of Bax and Bak also localize to the ER^14^, where they may affect cell survival via ER-derived Ca^2+^ signals^13,54^. Previous work by Hetz et al. further found that loss of Bax and Bak impaired IRE1α-dependent UPR signaling in MEFs and hepatocytes^28^. In pancreatic islets we observed the opposite; combined loss of Bax and Bak in Pdx1-expressing islet cells caused a moderate amplification of UPR-induced transcripts under glucolipotoxic conditions and following chemical induction of ER stress. It is possible that protection from ER stress-induced apoptosis spares a population of DKO cells that remain in the islet to cause an apparent amplification of the UPR. However, significant genotype-dependent differences in UPR transcripts are prominent at early stages of stress where β-cell death is minimal, which suggests Bax and Bak also dampen islet-cell ER stress and/or UPR signaling. Since the early UPR serves an adaptive role^38,55^, this raises the possibility that Bax and Bak may impair the β-cell’s ability for early ‘glucolipoadaptation’ in addition to facilitating late-stage death^41,56^. Differentiating between these outcomes will require careful consideration of the time- and context-dependent transition from adaptive to apoptotic β-cell UPR signaling.

In summary, we used mouse models of inducible and conditional gene deletion to dissect the roles of pro-apoptotic Bax and Bak in pancreatic β-cells. Our findings show that Bax and Bak do not play roles in normal β-cell function. Further, we provide new insights into the mechanisms by which glucolipotoxic β-cell death is executed, and show for the first time that these pro-apoptotic Bcl-2 family proteins can modulate ER stress responses in the endocrine pancreas.

## Methods

### Mice and in vivo studies

To establish the 4 genotypes of mice used for these studies we first bred Bax^flox/flox^:Bak^−/−^ mice (Stock number 006329, The Jackson Laboratory, B6:129 genetic background) with Pdx1-CreER mice (CD-1 genetic background)^57^ to create littermate mice with Bak single knockout (Bak SKO; Bax^flox/flox^:Bak^−/−^) and Bax-Bak double knockout (Bax-Bak DKO; Bax^flox/flox^:Bak^−/−^:Pdx1-CreER^+^)^30,58^. We further mated Bax^flox/flox^:Bak^+/−^ progeny to re-introduce the wild-type Bak allele and obtain a parallel colony of Bax single knockout (Bax SKO; Bax^flox/flox^:Bak^+/+^:Pdx1-CreER^+^) and littermate wild-type mice (WT; Bax^flox/flox^:Bak^+/+^). Whenever possible, littermates were compared (Bak SKO vs Bax-Bak DKO, and Bax SKO vs WT). In all other instances age- and sex-matched mice from the two parallel lines were used. To induce Bax gene deletion, tamoxifen (3 mg/40 g body weight) was administered daily by intraperitoneal (ip) injection for 4 consecutive days. The tamoxifen was freshly dissolved in warmed corn oil to 10 mg/ml followed by filter sterilization (millipore, steriflip 0.22 µm PES membrane) prior to injection. All mice, including controls, were injected with tamoxifen. In vivo glucose tolerance was assessed by ip glucose tolerance tests after a 6 h fast. Glucose (2 g/kg body weight) was administered by ip injection and tail vein blood droplets were read using OneTouch Ultra Blue Test Strips (Lifescan) at the indicated time-points. To minimize any putative long-term effects of Bax-Bak deletion, all in vivo experiments and islet isolations were done no later than 14 days following the final tamoxifen injection. The animal studies were approved by the University of British Columbia Animal Care Committee (animal protocol ID A16-0102) and carried out in accordance with the Canadian Council on Animal Care guidelines.

### Pancreatic islet isolation, dispersion, and culture

Mouse pancreatic islets were isolated from 12–16 week old mice by collagenase digestion followed by filtration-based purification, as previously described^30^. Isolated islets were hand-picked and cultured overnight before further treatment. Unless otherwise indicated, islets were cultured in RPMI 1640 completed with 2% penicillin–streptomycin and 10% fetal bovine serum (Gibco, Thermo Fisher). For experiments involving lipotoxic treatments, palmitic acid-containing culture media was prepared as detailed previously^1^. Briefly, a 20 mM stock solution was created by dissolving palmitic acid in 0.03 M NaOH, and this stock was used to prepare the final media containing 1,500 µM palmitate solution in complex with 20% fatty acid-free BSA in a 6:1 palmitate:BSA ratio. Control media contained similar amounts of BSA and NaOH. For single-cell analyses, islets were dispersed into single cells prior to study, as described^59^.

### Fluorescence microscopy

Glucose-induced changes in cytosolic Ca^2+^ were compared in cultured intact islets using the ratiometric fluorescent Ca^2+^ indicator fura-2, as described^59^. For measurements of mitochondrial membrane potential, dispersed islet cells were loaded for 30 min with 50 nM of the fluorescent indicator tetramethylrhodamine ethyl ester perchlorate (TMRE). Loading and imaging was done in phenol red-free RPMI 1640 and differences in mitochondrial membrane potential quantified by the absolute TMRE fluorescence intensity collected using a 585/60m emission filter following excitation using a 530/20× filter (Chroma Technology, Bellows Falls, VT, USA).

### Islet insulin secretion and content

For static assays of glucose-stimulated insulin secretion size-matched islets were first pre-incubated for 1 h in Krebs–Ringer Buffer (KRB; 129 mM NaCl, 4.8 mM KCl, 1.2 mM MgSO_4_, 1.2 mM KH_2_PO_4_, 2.5 mM CaCl_2_, 5 mM NaHCO_3_, 10 mM HEPES, 0.5% bovine serum albumin) containing 3 mM glucose. The islets were then incubated in KRB with 3 mM glucose and then 20 mM glucose for 1 h each, followed by 30 min incubation with KRB containing 3 mM glucose + 30 mM KCl. Supernatant was collected after each stimulation and assayed for secreted insulin. For quantification of insulin content, size-matched islets were washed twice with 1xPBS, collected in a 0.1 M HCl/70% ethanol solution and sonicated. Secreted insulin and insulin content were measured by radioimmunoassay (Rat insulin RIA Kit, Cedarlane, Burlington, ON, CA) or using the Mouse Ultrasensitive Insulin ELISA kit (ALPCO, Salem, NH, USA).

### Cell death assays

For quantification of cell death, dispersed islet cells were seeded into 96-well plates (Perkin Elmer ViewPlates) and allowed to adhere for 48 h in complete media. As previously detailed^30^, the cultures were stained with Propidium Iodide (0.5 μg/ml) and Hoechst 33342 (0.05 μg/ml) for 30 min before exposure to the indicated stress or control conditions. The 96-well plates were placed in an environmentally controlled (37 °C, 5% CO_2_) ImageXpress Micro high content screening system (Molecular Devices) for the duration of imaging. Subsequently, cell death was determined as the number of PI positive cells relative to the number of Hoechst 33342 positive cells using the MetaXpress software (Molecular Devices, San Jose, CA, USA).

### Real-time PCR analysis of islet mRNA

Total islet mRNA was extracted using the RNEasy Mini Kit (Qiagen) and cDNA was synthesized by reverse transcription using 100 ng RNA and the qScript cDNA synthesis kit (Quanta Biosciences). Target gene expression was measured relative to mouse β-Actin housekeeping gene using PerfeCTa SYBR Green SuperMix plus ROX (Quanta Biosciences) and assayed using Applied Biosystems StepOnePlus and Applied Biosystems 7500Fast Real-Time qPCR machines. Primers were synthesized from IDT (Table 1). The specificity of all primer pairs was assessed using BLAST and confirmed by the presence of single agarose gel bands at the expected size, as well as the presence of single melt curve peaks in the qPCR assays. Serial dilutions were used to validate that all amplification efficiencies ranged between 85 and 105%.

**Table 1:** Quantitative PCR primer sequences.

Gene	Forward (5′–3′)	Reverse (5′–3′)
*Actin*	GATCTGGCACCACACCTTCT	GGGGTGTTGAAGGTCTCAAA
*Atf6*	TGCCTTGGGAGTCAGACCTAT	GCTGAGTTGAAGAACACGAGTC
*Bak*	CTTTGGCTACCGTCTGGC	CAACCGCCTCTCTGTGCGA
*Bax*	GGAGCAGCTTGGGAGCG	AAAAGGCCCCTGTCTTCATGA
*Ddit3/Chop*	CTGCCTTTCACCTTGGAGAC	CGTTTCCTGGGGATGAGATA
*Dnajc3/P58ipk*	TCCTGGTGGACCTGCAGTACG	CTGCGAGTAATTTCTTCCCC
*Edem*	AAGCCCTCTGGAACTTGCG	AACCCAATGGCCTGTCTGG
*Erdj4*	TAAAAGCCCTGATGCTGAAGC	TCCGACTATTGGCATCCGA
*Ero1*	TCAGTGGACCAAGCATGATGA	TCCACATACTCAGCATCGGG
*Hspa5/Bip*	TCATCGGACGCACTTGGAA	CAACCACCTTGAATGGCAAGA
*Ins1*	GAAGTGGAGGACCCACAAGTG	ATCCACAATGCCACGCTTCT
*Ins2*	GAAGTGGAGGACCCACAAGTG	GATCTACAATGCCACGCTTCT
*Ire1*	CCGAGCCATGAGAAACAAGAA	GGGAAGCGGGAAGTGAAGTAG
*Pdia6/Pdip5*	AGCTCGTCAAGGATCGCCT	TATCACCTCTGCCCTGCTTTC
*Wfs1*	CGGGAAGAAACGGACAGAGC	CGTAGGTAGTGTTTGCCCAC
*Xbp1s*	GAGTCCGCAGCAGGTG	GTGTCAGAGTCCATGGGA

### Reagents and compounds

InSolution™ Staurosporine was from Calbiochem/EMD Millipore. Dimethyl Sulfoxide (DMSO), Thapsigargin (Tg), Palmitic Acid (PA), Propidium Iodide (PI), Tetramethylrhodamine Ethyl Ester Perchlorate (TMRE), Collagenase Type XI from *clostridium histolyticum*, corn oil and Tamoxifen (TM; Cat# T5648) were from Sigma-Aldrich (St. Louis, MO, USA). Fura-2/AM and Hoechst 33342 were from Invitrogen/Life Technologies (Burlington, ON, CA).

### Statistical analysis

Data are presented as mean ± SEM. Data analysis was performed using GraphPad Prism 6.0 software. Statistical analysis was performed by student’s t-tests, 1-way ANOVA, or 2-way ANOVA with Bonferroni post-hoc comparisons where appropriate. Differences were considered significant if p < 0.05.
